# Using digital technologies to engage with medical research: views of myotonic dystrophy patients in Japan

**DOI:** 10.1186/s12910-016-0132-2

**Published:** 2016-08-24

**Authors:** Victoria Coathup, Harriet J. A. Teare, Jusaku Minari, Go Yoshizawa, Jane Kaye, Masanori P. Takahashi, Kazuto Kato

**Affiliations:** 1 Centre for Health, Law and Emerging Technology, Nuffield Department of Population Health, University of Oxford, Ewert House, Banbury Road, Oxford, OX2 7DD UK; 2 Department of Biomedical Ethics and Public Policy, Graduate School of Medicine, Osaka University, 2-2 Yamadaoka, Suita, 565-0871 Osaka Japan; 3 Department of Neurology and Functional Diagnostics Graduate School of Medicine, Osaka University, D-4, 2-2 Yamadaoka, Suita, 565-0871 Osaka Japan

**Keywords:** Patient engagement, Dynamic consent, Electronic health records, Myotonic dystrophy, Digital technology, Participant centric initiatives, Patient privacy, Data sharing, Patient views, Patient perspectives

## Abstract

**Background:**

As in other countries, the traditional doctor-patient relationship in the Japanese healthcare system has often been characterised as being of a paternalistic nature. However, in recent years there has been a gradual shift towards a more participatory-patient model in Japan. With advances in technology, the possibility to use digital technologies to improve patient interactions is growing and is in line with changing attitudes in the medical profession and society within Japan and elsewhere. The implementation of an online patient engagement platform is being considered by the Myotonic Dystrophy Registry of Japan. The aim of this exploratory study was to understand patients’ views and attitudes to using digital tools in patient registries and engagement with medical research in Japan, prior to implementation of the digital platform.

**Methods:**

We conducted an exploratory, cross-sectional, self-completed questionnaire with a sample of myotonic dystrophy (MD) patients attending an Open Day at Osaka University, Japan. Patients were eligible for inclusion if they were 18 years or older, and were diagnosed with MD.

**Results:**

A total of 68 patients and family members attended the Open Day and were invited to participate in the survey. Of those, 59 % submitted a completed questionnaire (*n* = 40). The survey showed that the majority of patients felt that they were not receiving the information they wanted from their clinicians, which included recent medical research findings and opportunities to participate in clinical trials, and 88 % of patients indicated they would be willing to engage with digital technologies to receive relevant medical information. Patients also expressed an interest in having control over when and how they received this information, as well as being informed of how their data is used and shared with other researchers.

**Conclusion:**

Overall, the findings from this study suggest that there is scope to develop a digital platform to engage with patients so that they can receive information about medical care and research opportunities. While this study group is a small, self-selecting population, who suffer from a particular condition, the results suggest that there are interested populations within Japan that would appreciate enhanced communication and interaction with healthcare teams.

**Electronic supplementary material:**

The online version of this article (doi:10.1186/s12910-016-0132-2) contains supplementary material, which is available to authorized users.

## Background

Digital technologies are increasingly being applied in all areas of the patient pathway, both within research and the clinic, throughout healthcare systems across the world. Increasingly, the use of these technologies is focused on direct engagement with patients. The engagement opportunities of tools such as dynamic consent [1], a personalised electronic communication interface which enables people to give and change consent for involvement in research, are being realised in response to research participants' increasing support for greater interaction with research [2, 3].

In 2001, Japan launched the ‘e-Japan Priority Policy Program’ strategy, aimed at creating a society where everyone could benefit from IT in various different ways, including through the digitisation of health and medical care [4]. This has been implemented in a number of ways, including electronic medical records (EMR) [5] and the storage of patient data for research [6]. While this is promising, there has been little exploration of the value of such technologies in patient-facing processes.

The traditional doctor-patient relationship in the Japanese healthcare system has been of a paternalistic nature [7], which means it is not clear whether patients would welcome these initiatives or not. Higuchi conducted a comparison between Japanese and American doctor-patient interactions in the early 1990s and reported that deferring to doctors was consistent with people’s views of autonomy in Japan. In this study many Japanese patients did not accept the self-determined American style of consultation, but preferred to choose to stay uninformed and rely on the judgement of doctors [8]. However, in recent years there has been a gradual shift towards a more participatory patient-doctor model that allows a greater consideration of patient views in decision-making. Slingsby reported increasing active participation in the medical decision-making process, but also within the medical treatment process [9].

Despite this transition, there is still wide spread satisfaction with the traditional Japanese patient-doctor model [10], and a significant behaviour change may be required before digital solutions to support medical and research processes will extend to enable direct interaction with Japanese patients. What is not clear, is whether the bottleneck to enhance patient interaction is due to a lack of interest from participants, lack of provision, for cultural reasons or others. Given these unresolved questions, in instances where researchers are considering opportunities to set up electronic patient registries, it is not yet clear whether the adoption of technology to enable enhanced interaction between participants and the registry would be welcomed by participants, to allow greater control over their involvement in medical care and research. To explore this question, in contribution to the development of a registry for patients with myotonic dystrophy (MD), we have surveyed patients directly, to ascertain their views and attitudes to using an electronic patient registry to communicate with healthcare professionals and researchers.

## Methods

### Design

We conducted an exploratory, cross-sectional, self-completed questionnaire with a convenience sample of MD patients attending an Open Day at Osaka University, Japan. The Open Day was held in January 2015 and was organised by the Clinical Research Consortium for MD.

### Sample population

Patients were eligible for inclusion if they were 18 years or older, older, diagnosed with MD and attending an Open Day for patients at Osaka University Nakanoshima Center, Japan.

### Recruitment

The patient Open Day was advertised in two ways. Firstly, all MD patients who were attending outpatient clinics in Osaka University and Toneyama National Hospital, and met the eligibility criteria, were invited to attend by their clinician. Secondly, adverts were posted on hospital websites; they included details of the date, venue and outline for the patient Open Day. All patients attending the Open Day were invited to participate in the survey; each patient was given a questionnaire upon arrival to the Open Day and was invited to complete it at some point during the day. Researchers collected completed questionnaires as patients left the Open Day.

### Questionnaire design

The questionnaire was designed in a number of stages. Firstly, the initial themes were developed through discussions with all members of the research team, which included specialist clinicians and research scientists. Three themes were identified: current satisfaction with information from clinicians and researchers; current use of electronic devices; and acceptability of using electronic devices to engage with healthcare professionals and researchers. Secondly, MPT and KK developed a draft of specific sections of the survey, which was then modified by GY and JM. All members of the research team discussed the structure, content and language, and the questionnaire was redrafted a number of times, with careful consideration of the language use and meaning. The final draft consisted of a mixture of 16 closed and open-ended questions to gain a breadth and depth of patient views and attitudes, and took approximately 5 to 10 min to complete. A full copy of the questionnaire can be found as an additional file [see Additional file 1: Patient Survey in English.pdf]. To minimise social-desirability bias, the questionnaire collected no identifiable data from patients, ensuring anonymity. Consent was implied by the submission of a completed questionnaire.

### Analysis

Data was entered into a spreadsheet and participant responses were summarised as frequencies and percentages. Some participants did not answer all questions, therefore, the sample size varied by question. Percentages are calculated using total sample size (*n* = 40). Descriptive summary plots were generated to provide a visual representation of participants' responses to specific survey questions. Two-by-two tables were used to calculate proportions within specific strata of participants and Chi-squared test for independence was used to explore relationships between categorical questionnaire variables. Some questions permitted multiple responses. Therefore, some table responses do not add up to 40; these include questions on the type of information participants would like, what participants would like to tell doctors or researchers about, and what features of digital technologies would be important to participants. Analyses were performed using STATA 14.0 [11].

## Results

A convenience sample of 40 patients was recruited to the study. A total of 68 patients and family members attended the Open Day and were invited to participate in the survey. Of those, 59 % submitted a completed questionnaire (*n* = 40); one questionnaire was completed by a family member on behalf of the patient. All participants were Type 1 MD patients and the distribution of participant ages and gender are presented in Table 1. The majority of participants were females (62 %) and between the ages of 30 and 49 years old (56 %). Participants' age and gender were not associated with questionnaire responses.

**Table 1:** Gender and age of participants^a^ (*n* = 40)

	Number	Percent
Gender		
*Male*	10	24
*Female*	26	62
Age (years)		
*<20*	0	0
*20*–*29*	4	10
*30*–*39*	10	24
*40*–*49*	13	31
*50*–*59*	8	19
*60+*	3	7

^a^ *Patients only*

### Current communication with clinicians and researchers

Participants were initially asked to choose the areas in which they would like to receive more information from clinicians and researchers in the field. The most frequently reported areas of interest were associated with patients' disease state and prognosis, and information related to medical research. Of those who expressed an interest in learning more about medical research relating to MD, approximately 65 % of participants felt they either never received or received only a small amount of this information from clinicians or researchers. Results indicated there was no evidence of relationships between the types of information wanted, the amount of that information they currently receive or how satisfied participants are with day-to-day communication with their doctor (Table 2).

**Table 2:** The types of information participants would like to receive from doctors and researchers, the amount of information they currently receive and overall satisfaction with communication (*n* = 40)

	Receive information			Communication with doctor		
	All or some	Little or none	*p*-value	Satisfied	Dissatisfied	*p*-value
	*n* (%)	*n* (%)		*n* (%)	*n* (%)	
Types of information						
*Disease (general)*	8 (62)	5 (38)	0.390	12 (92)	1 (8)	0.058
*Symptoms*	8 (42)	11 (58)		14 (74)	5 (26)	
*Treatments*	9 (41)	13 (59)		16 (76)	5 (24)	
*Prognosis*	7 (28)	18 (72)		17 (74)	6 (26)	
*Other patients*	6 (46)	7 (54)		11 (85)	2 (15)	
*Medical research (findings)*	11 (35)	20 (65)		19 (68)	9 (32)	
*Medical research (participating)*	10 (43)	13 (57)		13 (62)	8 (38)	

Table 3 presents the type of information patients wish to tell their doctor about and how much of that information participants feel they are currently able to discuss. Results indicated that a higher proportion of participants felt they were able to discuss their physical conditions compared to issues about their mental health and lifestyle. Results also suggested that wanting to discuss issues relating to mental health or lifestyle was associated with a higher level of dissatisfaction with day-to-day communication with their doctor. Interestingly, satisfaction with day-to-day communication was associated with being able to tell doctors and researchers their issues, but was not associated with whether they received the information they were interested in (Table 4).

**Table 3:** The topics that participants would like to discuss with doctors and researchers, the amount participants currently discuss and overall satisfaction with communication (*n* = 40)

	Discuss information			Communication with doctor		
	All or some	Little or none	*p*-value	Satisfied	Dissatisfied	*p*-value
	*n* (%)	*n* (%)		*n* (%)	*n* (%)	
Important topics						
*Physical symptoms*	15 (60)	10 (40)	0.007	15 (60)	10 (40)	0.039
*Mental health*	5 (36)	9 (64)		7 (47)	8 (53)	
*Lifestyle/Household*	4 (24)	13 (76)		8 (44)	10 (56)

**Table 4:** The amount of desired information participants receive, topics discussed and overall satisfaction with communication (*n* = 40)

	Communication with doctor		
	Satisfied	Dissatisfied	*p*-value
	*n* (%)	*n* (%)	
Receiving information from doctor/researcher			
*All or some*	13 (54)	11 (46)	0.173
*Little or none*	10 (77)	3 (23)	
Discuss information with doctor/researcher			
*All or some*	15 (88)	2 (12)	0.002
*Little or none*	6 (37)	10 (63)	

### The use of digital technologies

Participants were then asked about their current use of digital technologies and whether they would be happy to register and engage with an online patient registry. Overall, participants expressed support for using computers, laptops or tablets to engage with medical care and research.

The majority of participants (73 %) reported using computers, tablets or smartphones on a daily basis, with reasons for use ranging from work (38 %), communicating with friends and family (48 %), and entertainment purposes (48 %).

Table 5 presents participants' current and future use of digital technologies. Approximately 70 % of participants reported using a computer or mobile terminal to answer questions or search for information about health or disease and 88 % reported they would be happy to use a computer or tablet to communicate with doctors or research health information in the future.

**Table 5:** Proportions of participants' use of digital technologies in healthcare (*n* = 40)

	Number	Percent
Have you ever used a computer or mobile terminal for collecting information about or answering questions about health or disease?		
*Yes, many times*	20	50
*Once or twice*	8	20
*Never*	11	28
Would you be happy to use a computer or tablet to communicate with a doctor or search for the types of information you are interested in?		
*Yes*	21	53
*Yes, but it would depend on certain conditions*	14	35
*I would prefer not to*	3	8
*Definitely not*	1	3
What do you think are the three most important features of a computer or tablet device that enables patients to engage with doctors or research?		
*Easy to view and enter information*	11	28
*Making the content easy to understand*	14	35
*Ability to use smartphone or tablet*	3	8
*Family members able to enter information on your behalf*	6	15
*Providing prompt responses to questions*	7	18
*Your information and privacy is well protected*	18	45
*Providing advice on how to treat both physical and mental symptoms*	16	40
*Making it useful for progress in medical research, diagnosing and treating patients*	13	33

**Fig. 1:**
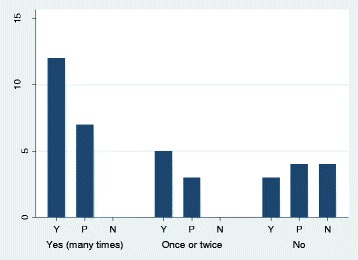
Frequency of participants who have reported they would use digital technologies to engage with healthcare in future (Y = Yes, very keen/*P* = Yes, depending on conditions/*N* = No), by frequency of participants' past use of computers, laptops and tablets to search for healthcare information (Yes (many times) / Once or twice / No)

Results indicated that participants who had used digital technologies to find out information about health or disease in the past were significantly more likely to use a computer or tablet to communicate with doctors or researchers in the future (X^2^ (4, *N* = 38) = 11.71, *p* = 0.020). Interestingly, of those who had never used digital technologies to find out about health or disease, approximately two thirds were interested in using computers or tablets in the future to communicate with their doctor or research the topics about MD they reported to be interested in; only four patients (10 %) reported no interest in using computers or tablets in relation to their disease in the future (Fig. 1).

When patients were asked about the three most important features of an electronic device that enabled patients to engage with doctors and research, the three most frequently reported features were: that their data is private and confidential (45 %); that it provides useful advice about treating both physical and mental symptoms of MD (40 %); and that the content is easy to understand (35 %). Interestingly, only 8 % of patients reported that the ability to access an application on their smartphone or tablet would be important to them (Table 5). Participants' choice of features were not associated with previous use of digital technologies or preferences for future use.

### Preferences for engaging with digital technology

Participants were also asked a number of questions regarding their views on receiving invitations to take part in medical research studies via an electronic patient registry. The majority of participants (68 %) stated they would like to receive emails about opportunities to participate in research; however, when asked about changing this preference, 75 % of all participants felt patients should be able to do this at any given time. The survey also explained that data from the patient registry might be shared with other researchers, including pharmaceutical companies. When asked about their preferences regarding data-sharing, the vast majority (78 %) of participants wanted to be informed in some way – either every time or at regular intervals – about how their data was being shared (Table 6).

**Table 6:** Participant attitudes to receiving information regarding opportunities to participate in medical research and participant data-sharing (*n* = 40)

	Number	Percent
Would you like to be informed about opportunities to participate in research via email or our website?		
*Yes*	27	68
*No*	2	5
*I don’t know*	8	20
Would you like to be informed every time your registry data is shared with other researchers or pharmaceutical companies?		
*I would like to be informed on every occasion*	17	43
*I would like to be informed at regular intervals*	14	35
*There is no need to inform me*	5	13
*I don’t know*	2	5

## Discussion

The main aims of this survey were to gauge patients’ interest in receiving information about medical research and to explore their views on using an electronic patient registry to communicate with healthcare professionals and researchers. The overall findings indicate that the MD patients (and in one case, family members) who participated in this survey were very interested in learning about medical research related to MD and would support the use of an electronic patient registry that they could use to engage with clinicians, find out more about their condition, and also discover opportunities to participate in medical research.

The findings from this survey suggest that currently the communication needs of patients, relating to research opportunities and general healthcare information, are not always being met. This is not necessarily unique to MD patients in Japan, as similar findings have been reported with other patient groups in other countries; for example in the UK a national survey conducted by the National Health Service (NHS) found that approximately 53 % of the cancer patients surveyed were given no information about relevant research and would have liked to be more informed about opportunities to participate [12].

The results also indicated that a higher proportion of participants felt they were not able to tell doctors or researchers about issues related to their mental health or lifestyle and household, compared to discussing physical symptoms. Being able to tell doctors or researchers about important issues was also associated with being satisfied with day-to-day communication with their doctor. Interestingly, receiving information was not associated with satisfaction. These results suggest that while this group of patients want information about their condition, they also value the opportunity to be listened to, and thus for a two-way communication channel. Similar findings were reported in a survey conducted in the UK with rare disease patients; only a third of respondents reported feeling like they received sufficient social and psychological support and approximately one quarter felt they receive adequate support with financial concerns related to their condition [13]. A lack of psychological support is of particular concern as there is evidence to suggest an association between suffering with MD and an increased risk of psychological conditions, such as depression and anxiety disorders, which can severely impact quality of life [14].

This suggests that a platform to enable greater levels of communication could be a positive improvement to the existing system, particularly given that several of the survey participants reported to have already used an electronic device to search for health information themselves. One possibility would be to use a digital communication system based on the concept of dynamic consent in which patients are connected with research projects and can access a record of their consent choices relating to the use of their samples and data, which they can change over time, if they wish to. Through such a platform they could also receive information about the research project and how it is progressing [1]. Furthermore, the digital system could pave the new way to facilitate communication of research participation within a family and develop the trust between the family and professionals [3].

The positive response to questions relating to the use of tablets, smartphones and computers to access health-related information, and the demonstration that a large proportion of this patient group are already confident in using technology for this purpose indicates that it is worth exploring this opportunity further. It is also interesting to note that while participants are interested in engaging through digital means, they would want to maintain control over this interaction; the majority of patients indicated they would like to be informed when their data are being shared for research purposes and also to have control over when and how they receive information about opportunities for research participation via email or website updates. This is particularly surprising given how much of a shift it is from the historical viewpoint of uninformed autonomy. Based on these results, it may be appropriate to consider implementing a platform that would enable continued engagement and communication with participants.

Despite the positive responses to using technology in healthcare, patients indicated that privacy was an important factor when using healthcare applications. Interestingly, a study conducted in the US reported that only a minority of patients had concerns over privacy issues in relation to healthcare technology and linked web messaging with clinicians [15]. A systematic literature review that summarised 12 studies conducted in North America and Europe, also reported that privacy did not appear to be a significant concern for patients [16]. However, patients included in the systematic review studies were from the general population, rather than rare disease patients. This, along with cultural differences, may account for the difference in attitudes towards privacy. Traditionally, there are negative implications associated with genetic disorders; inherited conditions were considered to bring shame upon a family [17, 18]. While this attitude has been changing over the past few decades, with more widespread access and acceptance of genetic counsellors, there still remains discrimination and prejudice regarding genetic disorders, such as MD [19, 20]. There are also a number of examples of projects incorporating health data that have been thwarted as a result of privacy concerns relating to data usage. One example is the care.data NHS initiative in the UK, which aimed to extract patients' medical data and share it with a central database using an opt-out system. The poor communication of this initiative has resulted in widespread public and professional concern over the privacy and control that patients have over their medical data [21]. Nonetheless, there is evidence to suggest that even those with higher than average privacy concerns are supportive of electronic platforms to share health data in the right context [22].

The patient views reported in the survey findings are consistent with the idea that there is a shift in Japanese culture and patients want to take more of a participatory role in their healthcare choices [9]. The interest demonstrated by this group may be related to their status as MD patients, and thus be in response to their general interest in learning more about their condition and how it influences their quality of life. Further research with alternative patient groups and healthy populations will be necessary to determine whether this standpoint is reflective of the general public, or specific to this interested group.

### Strengths and limitations

This study has a number of strengths. Primarily, it is the first survey to explore patients' views on using an electronic patient registry to communicate with healthcare professionals and researchers in Japan. Furthermore, the questionnaire was anonymous and self-completed; there is evidence to suggest that observational health research studies are particularly vulnerable to social-desirability bias in Japan, which has been attributed to the traditionally conservative Japanese culture [23]. By maintaining anonymity, we hope to have obtained more honest responses. While many of the respondents were women, a broad range of age groups were represented by the survey, ensuring that respondents weren’t just from a particularly technologically-savvy generation.

However, there are also a number of limitations that must be discussed. Firstly, the study recruited a small, self-selecting group of patients that had volunteered to take part in the patient Open Day, and thus were already demonstrating an interest in healthcare and research communication. The findings of this survey therefore may not be reflective of Japanese society in general, but do suggest that there are interested populations within Japan that would appreciate enhanced communication and interaction with healthcare teams. Therefore, the findings from this pilot study indicate that it would be appropriate for more work to be conducted to explore these issues in a wider population, including citizens and other patient groups. Secondly, while it would have been interesting to collect further socio-economic information from the participants, for example relating to education and income, there was a possibility that due to the small sample size attending the Open Day, patients may have been identifiable from their responses.

## Conclusions

Overall, the findings from this survey have demonstrated that many MD patients in Japan may be dissatisfied with the current communication with clinicians and other healthcare professionals and would welcome a digital platform to engage with clinical and research teams and learn more about medical research and opportunities to participate. The significant interest shown in both receiving information, and in making decisions about involvement, use of data and the types of information they receive was of particular interest, given how different this is from the widespread perceptions of Japanese healthcare and the role of patients.

The initial results presented by this survey provide the foundation for a number of new lines of enquiry. Firstly, whether different patient groups report similar experiences and have similar interests in receiving more information. Secondly, whether electronic platforms already being rolled out in other countries could be applied in Japanese clinical and research settings. Finally, further exploration of the sorts of conditions that would need to be applied if data were to be used for research, and contributed electronically, and how much control participants would want on setting these conditions.

## Additional file

Additional file 1: Patient Survey in English. (PDF 92 kb)

## Abbreviations

EMR: Electronic Medical Records
MD: Myotonic Dystrophy
