# Supplementary material for: Using digital technologies to engage with medical research: views of myotonic dystrophy patients in Japan
Source: BMC Med Ethics. 2016 Aug 24;17(1):51. doi: 10.1186/s12910-016-0132-2 (PMC4995774; doi:10.1186/s12910-016-0132-2)
Supplement: Additional file 1: — Patient Survey in English. (PDF 92 kb) [file 12910_2016_132_MOESM1_ESM.pdf]

Request for your cooperation with a questionnaire about changing patient registration to an Internet-based system

Research into treatments for neuromuscular diseases, notably myotonic dystrophy, is progressing, and moves are underway to start clinical trials. However, as few patients have these diseases, it would be difficult to find patients who meet the requirements for a clinical trial. There is also not enough information about matters such as the course of these diseases, the treatment patients are currently receiving and the effects of this treatment. Because of this, in recent years, a system of patient registration has been adopted.

We would like you to carry out the registration process yourself, so that you can tell us personal information such as your name and address. We currently register myotonic dystrophy patients by requiring patients to fill in a form and post it to the Department of Neurology and Cerebrovascular Diseases of Osaka University Hospital, but we are planning to use online registration in the future. However, we do not have much information about patients' preferences for the method and content of registration. We also hope that the Internet can be used to connect patients with their doctor and with researchers. Because of this, we have decided to conduct a questionnaire asking patients about their preferences regarding online registration, to help with registration, and to strengthen the connections between patients and their doctors and researchers.

We hope we can ask for you cooperation with the questionnaire, which is anonymous.

- The research is being conducted jointly, by hospitals including the Sendai Nishitaga National Hospital, Toneyama National Hospital, Aomori National Hospital, National Hospital Organization Higashisaitama Hospital, National Hospital Organization Suzuka National Hospital, and the National Center of Neurology and Psychiatry, in addition to Osaka University Hospital.
- The research targets myotonic dystrophy patients who are making outpatient visits, or who participate in patient groups or citizens' public lecture courses
- Cooperation with this research (replying to the anonymous questionnaire) is voluntary, and you will not suffer any disadvantage if you do not cooperate
- As the questionnaire is anonymous, no personal information will be used.
- Your response to the questionnaire may be sent to facilities conducting the joint research.
- As the questionnaire is anonymous, you cannot withdraw your response after you submit it.

- The results of research based on the results of the questionnaire may be published at venues such as academic conferences.
- After the end of the research (scheduled for the end of 2017), questionnaire forms will be stored for a certain period, and then disposed of.

December 2014

Professor Kazuto Kato, Department of Biomedical Ethics and Public Policy, Graduate School of Medicine, Osaka University
Investigator: Masanori Takahashi, Department of Neurology and Cerebrovascular Diseases, Osaka University Hospital

Inquiries: 2-2 Yamadaoka, Suita, Osaka 565-0871, Japan (Tel: 06-6879-5111)

Questionnaire

1. Is there anything you would like to ask doctors or researchers, or anything you would like them to tell you about? Choose any of the following that apply. (You can choose multiple answers.)

( ) Information about disease in general
( ) Information about the symptoms of your disease
( ) The tests or treatment plans for your disease
( ) The future course of your disease
( ) The state of other patients
( ) The latest information about medical research, including clinical trials
( ) Information about calls for participation in medical research, including clinical trials
( ) Other ( )

2. How much of the information that you want to know or be told about in 1 above do you normally receive?

( ) Almost everything
( ) Not everything, but some amount
( ) Only a small amount
( ) Nothing at all

3. Is there anything you would like doctors or researchers to know, or anything you would like to tell them?

( ) About your physical condition (that, if anything, it is good)
( ) About your physical condition (that, if anything, it is poor)
( ) About your mental condition (that, if anything, it is good)
( ) About your mental condition (that, if anything, it is poor)
( ) About your lifestyle (for example, financial matters or work-related matters)
( ) About your household
( ) Other ( )

4. How much of the information that you want doctors or researchers to know or want to tell them about in 3 above are you normally able to communicate?

( ) Almost everything
( ) Not everything, but some amount
( ) Only a small amount
( ) Nothing at all

5. How satisfied are you with your day-to-day communication with your doctor?

( ) Very satisfied
( ) Somewhat satisfied
( ) Not very satisfied

( ) Almost entirely dissatisfied

See reverse side

6. Could you tell us what areas should be improved?

[ ]

7. When patients are registered, we ask whether they want to receive information about clinical trials. We are considering making it easier to change this stated preference. What do you think about this?

( ) There is no need to change it.

( ) It should be possible to change it whenever you want.

( ) It should be possible to change it only when the information is updated once per year.

( ) Other ( )

8. There may be new researchers who want to study specific symptoms of your disease (such as difficulty swallowing) in detail. Would you like us to inform you about participation in this research via your registered e-mail address or our website?

( ) Yes

( ) No

( ) I don't know.

9. Data on registered patients which includes personal information may be provided to parties such as researchers or pharmaceutical companies if they make an application to the committee and are approved. Would you like us to inform you of the specific details of how your data has been provided to outside parties?

( ) I would like to be informed on each occasion.

( ) I would like to be informed at regular intervals.

( ) There is no need to inform me.

( ) I don't know.

10. Have you ever used a computer or mobile terminal for collecting information about, or for questions and answers about, health or disease?

( ) Yes, many times

( ) Yes, once or twice

( ) No, never

11. Would you like to use a computer or mobile terminal to communicate with a doctor or research about the types of topics discussed in questions 1 to 9 above?

( ) Yes, I would be keen to do so.

( ) Yes, but it would depend on certain conditions.

( ) If anything, I would prefer not to.

( ) No, not at all.

12. This question is for everyone who did <u>not</u> answer "No, not at all." for question 11. What aspects do you think we should adapt, pay attention to or make effective? Please select the most suitable answers, up to a maximum of 3 answers.

( ) Making it easy to view and enter information

( ) Making the content easy to understand

( ) Enabling you to use a smartphone or tablet to enter information, rather than a computer

( ) Enabling you to have someone else, such as a family member, enter information

( ) Providing fast (early) answers and responses

( ) Making sure that your information and privacy is well protected

( ) Enabling you to obtain useful information for the care of your physical or mental state

( ) Making it useful for progress in medical research, and for diagnosing and treating other patients with the same disease

13. How often do you use computers or other information devices (such as smartphones or tablets)?

( ) Not at all

( ) Only very rarely

( ) About once a month

( ) About once a week

( ) Almost every day

14. What types of computers or information devices do you use? Please check all the answers that apply. (You can give multiple answers.)

( ) Desktop computers

( ) Notebook computers

( ) Tablet computers

( ) Smartphones

( ) Computer game consoles

( ) Other ( )

( ) I do not use any of these

15. What do you use computers or other information devices for? Please check all the answers that apply. (You can give multiple answers.)

( ) Work

( ) Tasks other than work

( ) Communication with other people (for example, Skype or Line)

( ) Entertainment (such as television or games)

( ) Other ( )

( ) I do not use computers or other information devices

16. Could you please circle your sex and approximate age?

Male Female

Under 20 20-29 30-39 40-49 50-59 60 or over

Thank you for your cooperation.
